# A Silver–Copper–Aluminum Layered Double Hydroxide Sensor for Sensitive Determination of Anticancer Agent Afatinib in Bulk and Biological Fluids

**DOI:** 10.1021/acsomega.5c06746

**Published:** 2025-09-30

**Authors:** Edoh Nicodème Gabiam, Nevin Erk, Mehmet Soner Bay, Asena Ayşe Genc, Hassan Elzain Hassan Ahmed, Mustafa Soylak

**Affiliations:** † 37504 Ankara University, Faculty of Pharmacy, Department of Analytical Chemistry, Ankara 06100, Turkey; ‡ 52958 Erciyes University, Faculty of Sciences, Department of Chemistry, Kayseri 38280, Turkey; § Erciyes University, Technology Research & Application Center (TAUM), Kayseri 38280, Turkey; ∥ Turkish Academy of Sciences (TUBA), Ankara 06100, Turkey

## Abstract

Afatinib (AFA), a powerful tyrosine kinase inhibitor, is an FDA-approved drug used to treat advanced nonsmall cell lung cancer (NSCLC) with certain EGFR mutations. As the first irreversible EGFR inhibitor approved for the treatment of lung cancer, it plays a key role in blocking EGFR signaling, making it a significant therapy in targeted cancer treatment. This study presents a pioneering electrochemical approach for determining AFA, a clinically significant anticancer agent, utilizing a novel sensor based on a trimetallic nanocomposite, silver–copper–aluminum layered double hydroxide (AgCuAl-LDH). The sensor was fabricated through a facile, cost-effective hydrothermal synthesis method, resulting in a robust and highly conductive nanomaterial. Structural and morphological characterization via X-ray diffraction (XRD) and scanning electron microscopy (SEM) confirmed the successful formation of the nanocomposite with desirable crystalline and surface properties. Electrochemical evaluation of AFA was conducted using cyclic voltammetry (CV) and differential pulse voltammetry (DPV), where the sensor exhibited a significantly enhanced response. Electrochemical impedance spectroscopy (EIS) further validated the superior electrochemical performance of the sensor, showing reduced charge transfer resistance and elevated conductivity. The proposed sensor demonstrated outstanding analytical performance with a high sensitivity of 1.65 μA·μM^–1^·cm^–2^, a wide linear detection range from 0.02 to 13.1 μM, and an impressively low detection limit of 2.99 nM. Importantly, the sensor was successfully applied to real pharmaceutical formulations and biological samples, confirming its practical utility in clinical and quality control settings. This work marks the first electrochemical detection strategy for Afatinib, filling a critical gap in analytical methodologies and paving the way for advanced, efficient, and accessible sensing platforms in oncology drug monitoring.

## Graphical abstract

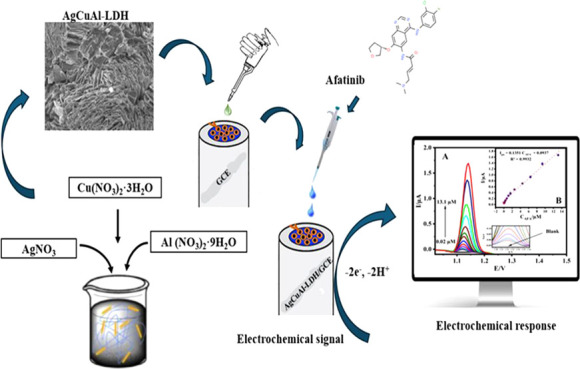

## 1 Introduction

In the realm of electrochemical sensing, the quest for enhanced sensitivity, selectivity, and stability remains paramount for developing efficient sensing platforms capable of detecting various analytes with precision and reliability. Among these analytes, anticancer agents hold particular significance due to their critical role in combating cancer, a disease that continues to pose significant challenges to global health. Anticancer drugs, while crucial for the treatment of various malignancies, pose significant environmental hazards. These pharmaceuticals often persist in the environment due to their complex chemical structures and resistance to biodegradation.^1^ Wastewater treatment plants are not fully effective at removing these compounds, leading to their discharge into aquatic systems.^2,3^ Once in the environment, anticancer drugs can have detrimental effects on aquatic life, disrupting endocrine systems and affecting reproductive health in fish and other organisms. Additionally, the accumulation of these drugs in the environment can contribute to the development of drug-resistant microorganisms, posing a broader ecological and public health risk.^4^ Therefore, the detection and monitoring of anticancer drugs in environmental samples are of paramount importance to mitigate their adverse effects.

Afatinib (AFA), (E)-N-[4-(3-chloro-4-fluoro-anilino)-7-[(3S)-tetrahydrofuran-3-yl]­oxy-quinazolin-6-yl]-4-(dimethylamino)­but-2-enamide (Figure 1), a potent anticancer agent belonging to the class of tyrosine kinase inhibitors, has emerged as a vital therapeutic option for treating advanced nonsmall cell lung cancer (NSCLC) with specific epidermal growth factor receptor (EGFR) mutations. It is the first FDA-approved irreversible inhibitor specifically authorized for the treatment of lung cancer.^5^ Its effectiveness in inhibiting EGFR signaling pathways underscores its clinical importance. However, the precise and sensitive detection of AFA in biological fluids and pharmaceutical formulations is crucial for optimizing therapeutic regimens, monitoring drug levels, and preventing potential adverse effects associated with either suboptimal dosing or overdose.

**Figure 1:**
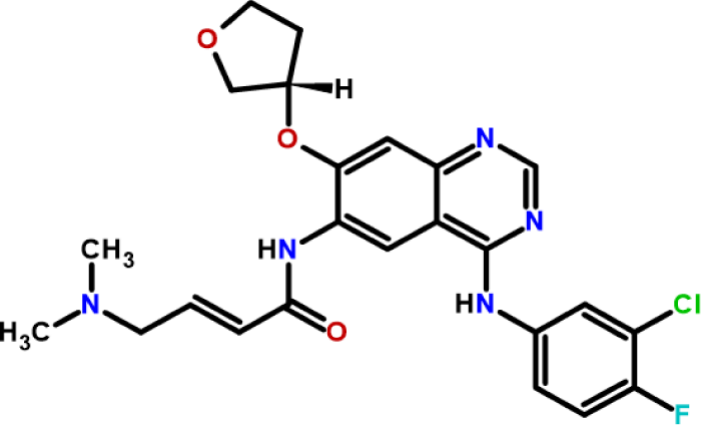
Chemical structure of afatinib.

In this context, the development of advanced electrochemical sensing platforms holds promise for addressing the challenges associated with AFA detection. Among the various materials explored for sensor fabrication, layered double hydroxides (LDHs) have garnered significant attention. LDHs, characterized by their tunable composition, high surface area, and excellent electrochemical properties, offer an ideal scaffold for designing efficient sensing platforms.^6^

In recent years, the synthesis of LDH-based nanocomposites incorporating noble metals such as silver (Ag) and bimetallic combinations (e.g., AgCu, AgAl, and AgCuAl) has emerged as a promising strategy to enhance the performance of electrochemical sensors.^7,8^ The incorporation of noble metals not only facilitates electron transfer processes but also enhances catalytic activity, leading to improved sensor sensitivity and selectivity.

Numerous methods, including coprecipitation,^9^ sol–gel,^10^ and green synthesis,^11^ have been used for the synthesis of LDH-based nanocomposites. The coprecipitation method is popular for its simplicity and cost-effectiveness, allowing easy composition control and suitability for large-scale production, but often yields materials with lower crystallinity requiring further treatment.^12^ The sol–gel method provides precise control over material composition and homogeneity, ideal for complex oxides and hybrids, but involves longer processing times and higher costs for large-scale production.^13^ Green synthesis uses natural sources and biological agents, minimizing toxic chemicals and environmental impact, yet faces challenges in scalability and consistency of material properties.^14^

Hydrothermal synthesis stands out as a versatile and efficient technique for fabricating LDH-based nanocomposites with precise control over composition, morphology, and structure.^15,16^ By manipulating synthesis parameters, such as temperature, pressure, and precursor concentrations, it becomes possible to tailor the characteristics of LDH-based materials for specific sensing applications.

To date, numerous techniques utilizing high-performance liquid chromatography (HPLC)^17^ and liquid chromatography–tandem mass spectrometry (LC–MS/MS)^18–20^ have been developed to quantitatively analyze AFA in different types of samples, either on its own or in conjunction with other pharmaceutical compounds. However, many of these methods are costly, require extensive time and effort, and are inconvenient due to their complexity and the time-consuming nature of the experimental procedures.^21^ Such limitations can hinder their applicability, especially in routine and point-of-care diagnostics.

In contrast, electrochemical techniques have emerged as a compelling alternative, offering rapid response, operational simplicity, cost-effectiveness, and high analytical sensitivity for the detection of biologically relevant molecules.^22^ These attributes make them particularly attractive for real-time analysis and clinical applications. Notably, despite the extensive research conducted on Afatinib quantification, a thorough survey of the current scientific literature reveals a conspicuous absence of studies employing electrochemical strategies for its determination. This significant research gap highlights both the novelty and the necessity of the present work.

In this study, we report for the first time the electrochemical detection of Afatinib using a sensor based on hydrothermally synthesized AgCuAl-LDH nanoparticles. The sensor exploits the synergistic interplay between the catalytic activity of incorporated noble metals (Ag and Cu) and the high surface area, structural stability, and ion-exchange properties of the LDH matrix. This hybrid composition is designed to significantly enhance electron transfer kinetics and surface adsorption of Afatinib molecules, thereby enabling sensitive, selective, and reproducible electrochemical measurements. Our findings not only address the limitations of existing methods but also establish a foundational platform for developing next generation sensing systems with high performance and practical applicability in pharmaceutical quality control and clinical diagnostics.

## 2 Materials and Methods

### 2.1 Chemicals

For further insights and comprehensive details, please consult the Supporting Information section.

### 2.2 Synthesis of AgCu-LDH, AgAl-LDH, and AgCuAl-LDH Using the Hydrothermal Method

The hydrothermal technique was employed to synthesize AgCu-LDH, AgAl-LDH, and AgCuAl-LDH. For AgCu-LDH synthesis, AgNO_3_ (5 mM) and Cu­(NO_3_)_2_·3H_2_O (5 mM) were dissolved in 60 mL of distilled water, followed by the addition of 37 mmol of urea and 10 mmol of NH_4_F to the beaker. The solution was thoroughly mixed using a magnetic stirrer for 30 min. The mixture was then transferred to a stainless-steel autoclave lined with polytetrafluoroethylene (PTFE) and placed in an oven at 120 °C for 12 h. After allowing the autoclave to cool down naturally to ambient temperature, the AgCu-LDH sample was subjected to ultrasonic washing, multiple washes with absolute ethanol and deionized water, and finally, overnight drying in an oven at 70 °C.

Similar to AgCu-LDH, the synthesis of AgAl-LDH and AgCuAl-LDH also employed the hydrothermal method. For AgAl-LDH (AgNO_3_ and Al­(NO_3_)_3_·9H_2_O) and AgCuAl-LDH synthesis, equivalent amounts (moles) of AgNO_3_, Al­(NO_3_)_3_·9H_2_O, and Cu­(NO_3_)_2_·3H_2_O were dissolved in 60 mL of distilled water. Urea and NH_4_F were then added to the mixture, followed by thorough stirring for 30 min to ensure uniform mixing. The obtained solution was transferred to a stainless-steel autoclave lined with PTFE and placed in an oven at 120 °C for 12 h (Figure 2). For washing, the same protocol was utilized.^23,24^

**Figure 2:**
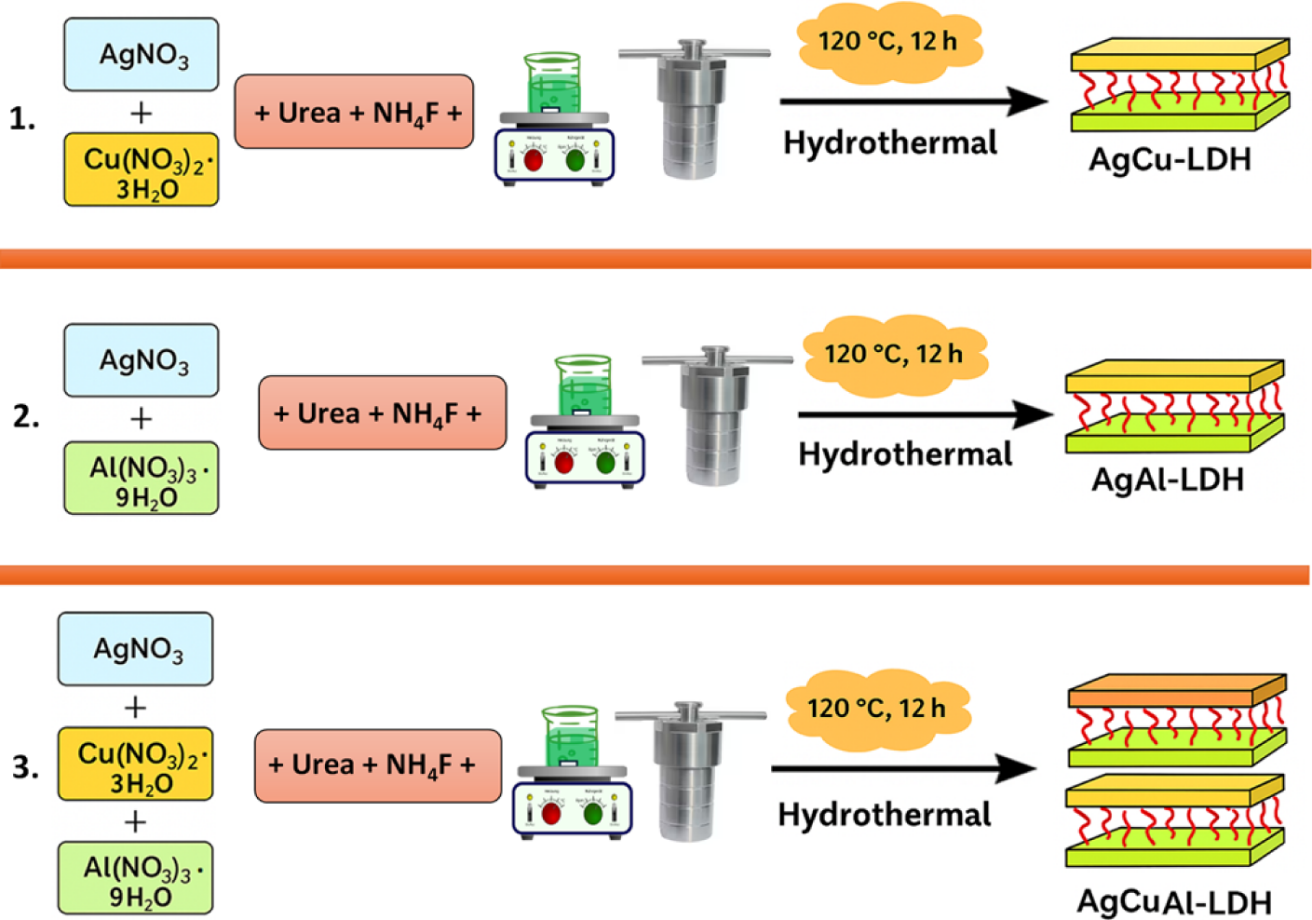
Schematic illustration of the hydrothermal synthesis process for AgCu-LDH, AgAl-LDH, and AgCuAl-LDH.

### 2.3 Fabrication of AgCuAl-LDH/GCE

First, the glassy carbon electrode (GCE) was polished with an alumina cleaning kit to achieve a reflective surface. Following this, any residue of alumina on the electrode’s surface was eliminated through sonication, followed by a meticulous rinse with deionized water, and finally dried. Subsequently, 1.0 mg of the AgCuAl-LDH was introduced into 1.0 mL of distilled water and then underwent ultrasonication for 90 min to attain a uniform suspension. To immobilize AgCuAl-LDH onto the tip of the GCE, the electrode was inverted, and 6 μL of the suspension solution of AgCuAl-LDH was carefully deposited onto the electrode surface using a micropipette. The pretreated GCE was then allowed to dry in the air for 15 min at room temperature. Consistent immobilization time was maintained across all experiments.

### 2.4 Real Samples Preparation

For the analysis of AFA content in tablets, ten Giotrif tablets containing 600 mg each were pulverized into a fine powder. Subsequently, a suitable quantity of powdered tablets was dissolved in 100 mL of water through ultrasonication to prepare the tablet solution. To analyze human serum, 1 mL of the serum sample was combined with 1 mL of acetonitrile to precipitate the serum proteins. The mixture was then centrifuged at 6000 rpm for 15 min. A suitable volume of the resulting supernatant was subsequently mixed with a standard solution of AFA to reach a final concentration of 1.0 μM.

The drug-free human urine used in this study was sourced from healthy volunteers and filtered utilizing a 0.45 μm PTFE syringe filter. Next, 5 mL of the filtered urine sample was thoroughly combined with 5 mL of AFA solution.

The determination of AFA in real samples was performed using the standard addition technique.^25,26^

## 3 Results and Discussion

### 3.1 Characterization of AgCu-LDH, AgAl-LDH, and AgCuAl-LDH

The SEM images depict three distinct types of LDHs: AgCu-LDH (a, b and c), AgAl-LDH (d, e and f), and AgCuAl-LDH (g, h and (i) (Figure 3). These LDH structures impart several intriguing properties, including a substantial surface area, efficient adsorption capacity, favorable electrochemical characteristics, and adjustable compositions. Figure 3a–c portrays a bimetallic LDH with a tetragonal plate-like form, evident in the SEM visuals. In Figure 3d–f, another bimetallic LDH exhibits a similar tetragonal plate-like shape but with a smaller average particle size of 200 nm compared to AgCu-LDH. AgAl-LDH has demonstrated effectiveness in eliminating toxic metals from wastewater and catalyzing chemical reactions. Figure 3g–i presents a trimetallic LDH containing Ag, Cu, and Al, displaying a more intricate structure than AgCu-LDH and AgAl-LDH, comprising clusters of small, flower-like nanoparticles. AgCuAl-LDH, with its considerable surface area, has proven valuable for applications such as catalysis and water treatment.

**Figure 3:**
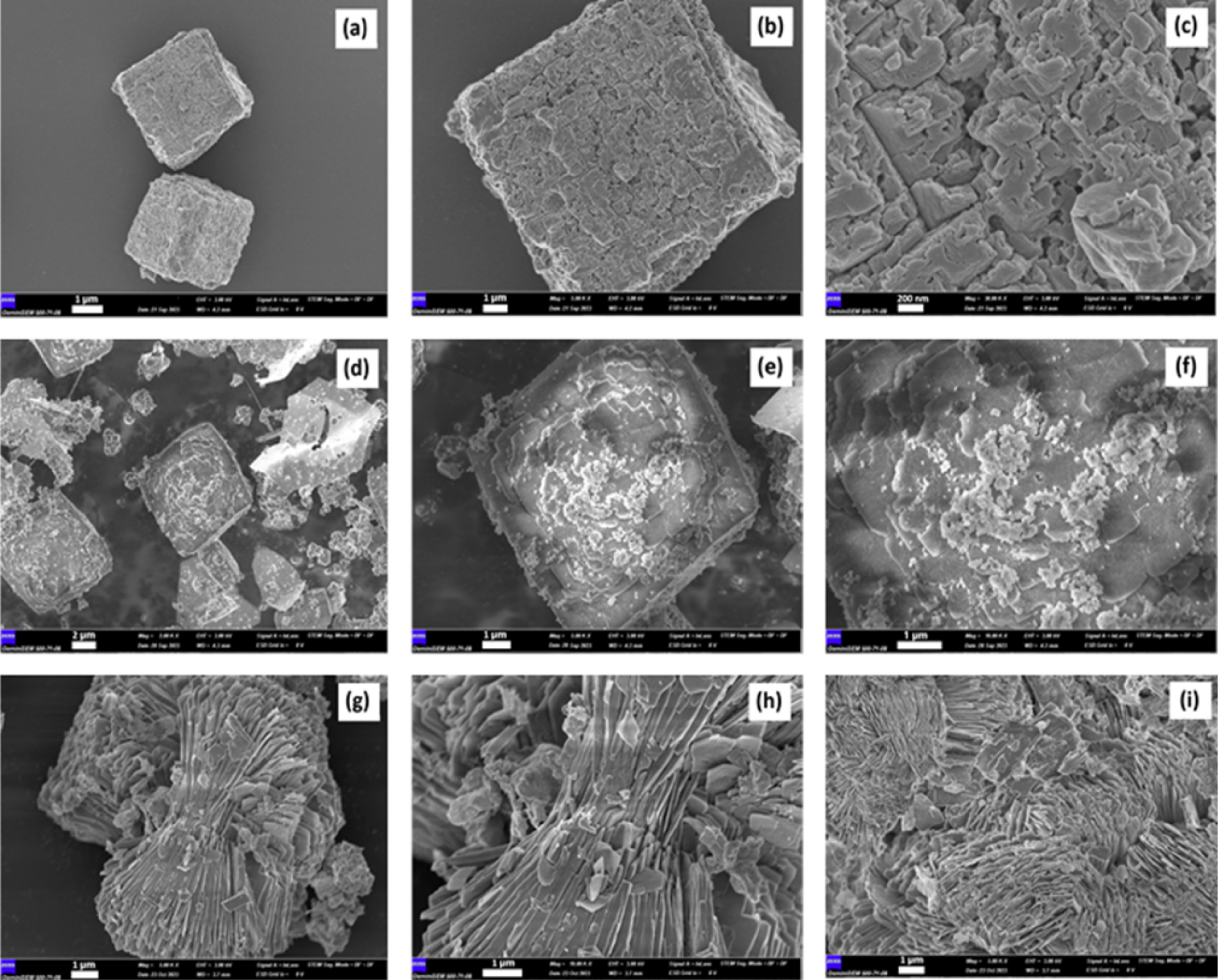
FE-SEM images of AgCu-LDH (a–c), AgAl-LDH (d–f), and AgCuAl-LDH (g–i).

Figure 4 presents the SEM-EDX analysis and elemental composition of the three newly synthesized LDHs. The elemental contents align with those known to be present in these LDHs.

**Figure 4:**
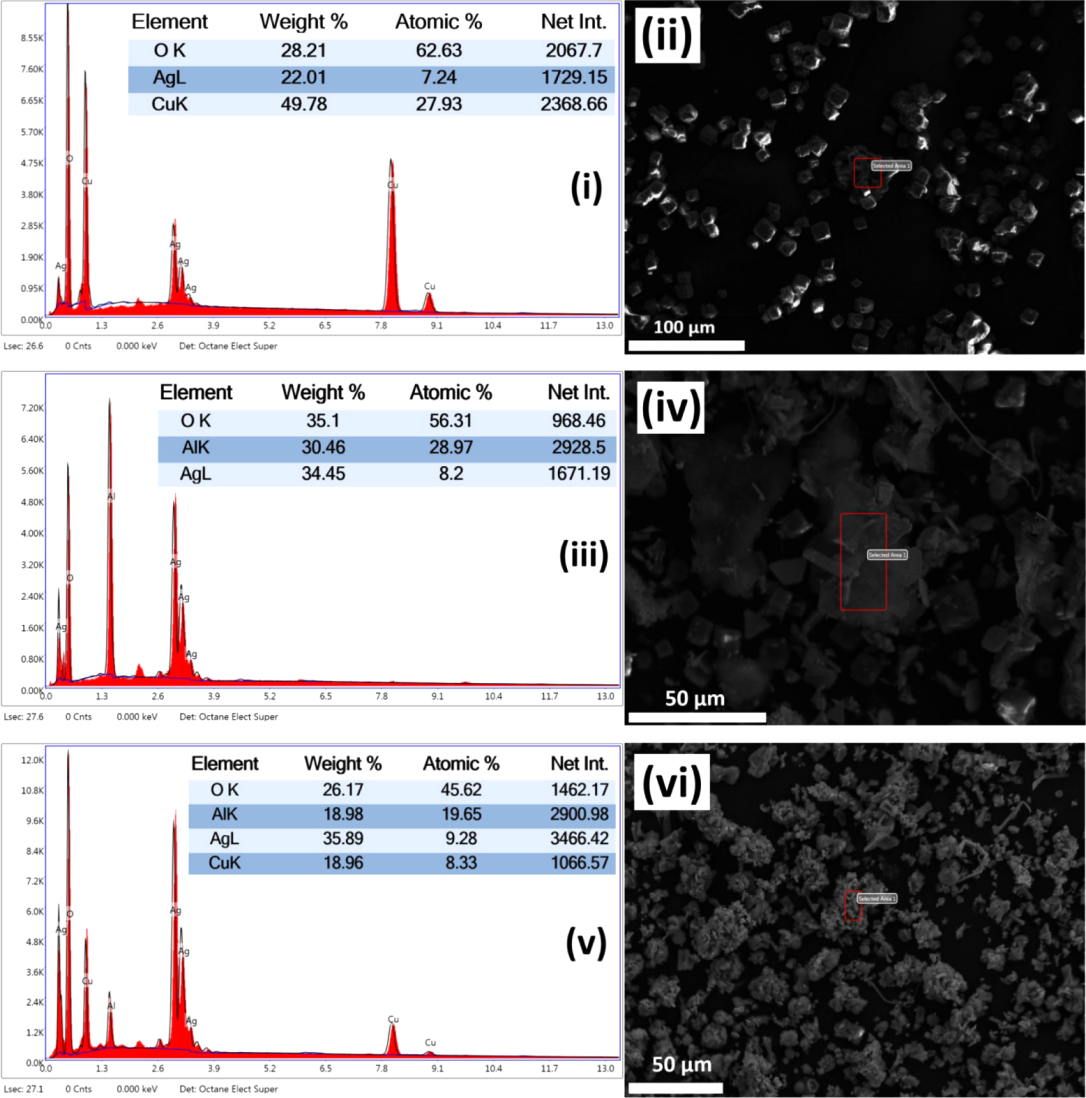
SEM-EDX analysis and element composition percentages of AgCu-LDH: (i) & (ii), AgAl-LDH: (iii) & (iv), and AgCuAl-LDH: (v) & (vi).

In the AgCu-LDH spectrum (i, ii), the high Cu content (49.78 wt %) along with Ag (22.01 wt %) and O (28.21 wt %) confirms the formation of a bimetallic LDH with Cu as a dominant metal species. The AgAl-LDH (iii, iv) sample exhibits a different elemental distribution, with a higher O content (35.1 wt %) and a significant presence of Al (30.46 wt %) and Ag (34.45 wt %). This indicates the successful combination of Al^3+^ into the LDH structure alongside Ag. In the ternary AgCuAl-LDH (v, vi), the balanced presence of Cu (18.96 wt %), Al (18.98 wt %), Ag (35.39 wt %), and O (26.17 wt %) confirms the effective cosubstitution of both Cu and Al into the layered structure. These results suggest the targeted stoichiometry and successful integration of metal ions into the LDH system.

The XRD patterns in Figure 5 show the presence of both AgCu-LDH and AgAl-LDH phases. This is evident from the diffraction peaks marked with (#), which are common to both phases. However, the presence of distinct diffraction peaks at 2θ values of 23.8, 37.2, 38.5, 43.7, 64.4, 77.8, and 82.1 confirms the formation of the AgCuAl-LDH phase. These peaks correspond to diffraction from basal planes (0 0 3), (0 0 6), (0 0 9), (0 1 2), (0 1 5), (0 1 8), and (1 1 0), respectively. The formation of the AgCuAl-LDH phase is likely due to the presence of Cu­(II) cations in the precursor solution. Cu­(II) cations have a higher affinity for the LDH layers than Ag­(I) cations, so they are preferentially incorporated into the structure. This leads to the formation of a ternary LDH phase containing Ag­(I), Cu­(II), and Al­(III) cations.^27^ The formation of mixed-metal LDH phases is often desirable, as they can exhibit improved properties compared to single-metal LDH phases. For example, mixed-metal LDH phases have been shown to have higher thermal stability and catalytic activity than single-metal LDH phases.^28^

**Figure 5:**
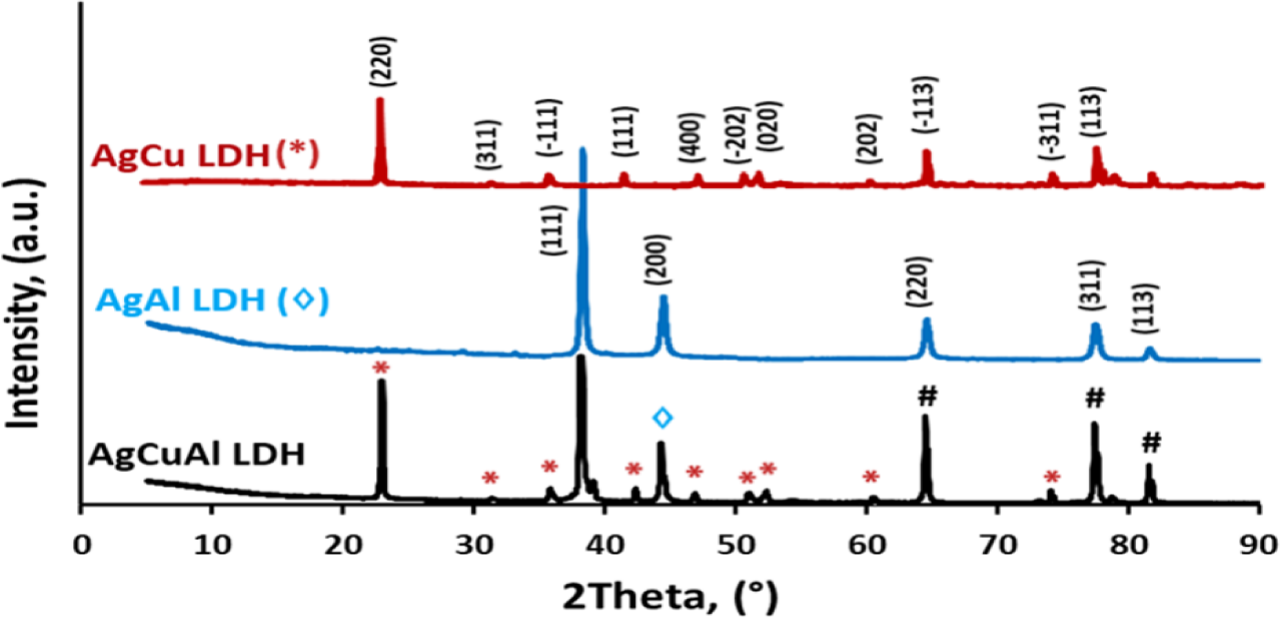
XRD patterns of AgCu-LDH, AgAl-LDH, and AgCuAl-LDH.

Figure 6 shows the FT-IR spectrum of AgCu-LDH (a), AgAl-LDH (b), and AgCuAl-LDH (c) within the wavelength range of 4000–500 cm^–1^. The spectra are characterized by two prominent transmittance bands at 3390, 2990, and 2902 cm^–1^. These bands correspond to the stretching vibrations of O–H (from water molecules) and C–H, respectively. The transmittance spectra at 2120 cm^–1^ and 1720 cm^–1^ indicate the presence of nitrile (CN) and carbonyl (C=O) groups in the compound. The transmittance bands at 1390, 1095, 860, and 550 cm^–1^ are attributed to O–H bending, C–O stretching vibration, C=C bending, and C-metals bonds, respectively. The presence of these diverse transmittance bands proves the effective synthesis of AgCuAl-LDH nanoparticles.^29–31^

**Figure 6:**
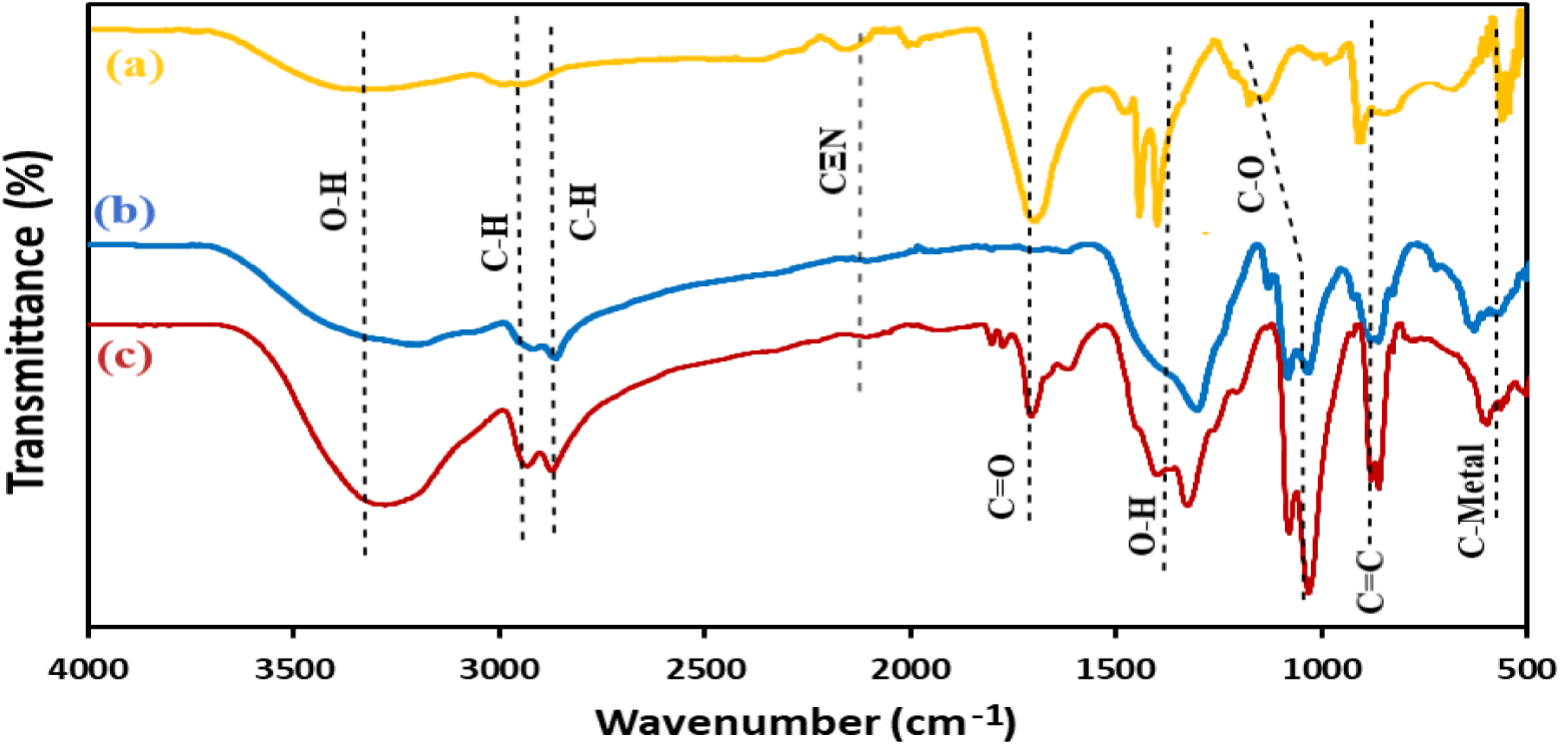
FT-IR spectrum of AgCu-LDH (a), AgAl-LDH (b), and AgCuAl-LDH (c).

### 3.2 Electrochemical Behaviors of Modified Electrode

The electrocatalytic efficiency of GCE modified with AgCu-LDH, AgAl-LDH and AgCuAl-LDH nanoplates, as illustrated in Figure S1, was assessed by DPV and CV responses in a B-R buffer solution containing 0.1 mM of AFA. The experiments were performed with and without AFA in the B-R buffer solution (0.1 mM, pH 1.0). As can be seen in Figure S1A, in the absence of AFA, the baseline voltammogram of the modified electrodes exhibited no oxidation peak, indicating the composites’ inert behavior. When 0.1 mM AFA was introduced, the unmodified GCE displayed an oxidation peak of 5.25 μA at 1.15 V. On the other hand, the modified electrodes showed prominent oxidation peaks at 1.14 V (vs Ag/AgCl), with a substantially enhanced current response of 7.35 V for the AgCu-LDH/GCE, 5.74 μA for AgAl-LDH/GCE, and 9.34 μA for AgCuAl-LDH/GCE. The findings demonstrate a significant enhancement in the electrochemical activity of AFA on the AgCuAl-LDH/GCE, as reflected by the observed negative shift in the AFA oxidation potential and the simultaneous rise in peak current. A similar pattern is observed in the CV results presented in Figure S1B, where the modified electrodes deliver the highest current response

Moreover, the CV method and electrochemical impedance spectroscopy (EIS) of 5.0 mM [Fe­(CN)_6_]^3‑/4–^ in 0.1 M KCl were measured on the surface of the AgCuAl-LDH nanoplate-modified GCE (Figure 7). Initially, the CV on the surface of the bare GCE (at a scan rate of 50 mV s^–1^) displayed the lowest current density (Bare/GCE). The application of the synthesized nanocomposites (AgCu-LDH, AgAl-LDH and AgCuAl-LDH onto the GCE electrode resulted in an increased current density, which can be attributed to a diminution in electron transfer resistance (Figure 7A).

**Figure 7:**
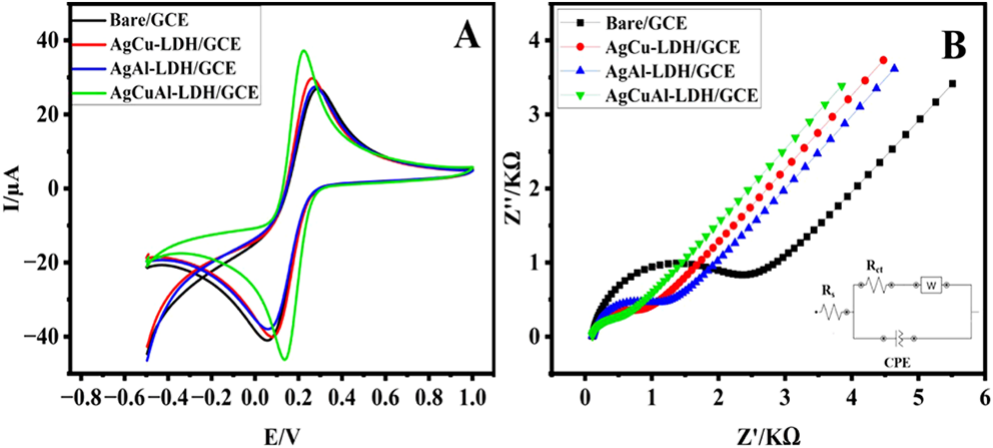
CV (A) and (EIS) (B) performed on the bare and modified electrodes in a solution containing 5.0 mM [Fe­(CN)_6_]^3‑/4–^ with 0.1 M KCl utilizing a scan rate of 50 mV s^–1^.

The peak potential separations obtained for the different electrodes demonstrated that the modified GCEs exhibited lower separation values compared to the bare GCE. This observation provides additional evidence of the enhanced electrochemical performance resulting from the integration of AgCuAl-LDH nanocomposites onto the GCE surface. The detailed values are summarized in Table 1.

**Table 1:** Electrochemical Parameters Obtained from CV and EIS for Various Working Electrodes

**Electrodes**	**ΔE**_**p**_ **(V)**	**ESA (cm** ^**2**^)	**K** ^**0**^ **(cm s** ^**–1**^)	**J**_**0**_ **(A cm** ^**2**^)
Bare/GCE	0.23	0.050	34.7 × 10^–5^	16.75 × 10^–5^
AgCu-LDH/GCE	0.20	0.082	42.29 × 10^–5^	20.40 × 10^–5^
AgAl-LDH/GCE	0.17	0.087	44.29 × 10^–5^	22.17 × 10^–5^
AgCuAl-LDH/GCE	0.14	0.120	55.22 × 10^–5^	26.64 × 10^–5^

The electrochemical properties of the pristine and modified electrodes were analyzed using EIS (Figure 7B). The conductivity and electron transport characteristics of the electrodes are reflected by the Rct value. A lower Rct value indicates higher conductivity and a faster rate of electron transfer from the electrode being analyzed.^32^ A considerable increase in electron transfer resistance (Rct) was observed at the unmodified GCE (3068.4 Ω) (Bare/GCE). In contrast, the incorporation of the developed nanocomposites onto the GCE surface resulted in a marked reduction in Rct, measured at AgCu-LDH/GCE (1535.5 Ω), AgAl-LDH/GCE (1331.5 Ω), and AgCuAl-LDH (803.73 Ω). The observed decrease in Rct for the synthesized AgCuAl-LDH nanocomposite is primarily ascribed to its enhanced electrical conductivity, along with the synergistic interaction among the Ag, Cu, and Al constituents, which collectively promote more efficient electron transfer processes.^33^

The specific surface areas of the untreated and modified electrodes were assessed employing the Randles-Sevcik equation (eq S1) alongside slope analysis, as depicted in Figure S2, wherein the square root of the scan rate was correlated with the peak current. The calculations yielded an electroactive surface area (ESA) of 0.050, 0.082, 0.087, and 0.120 cm^2^ for the untreated GCE AgCu-LDH/GCE, AgAl-LDH/GCE, and AgCuAl-LDH/GCE, respectively (Table 1). These results unambiguously demonstrate that AgCuAl-LDH, as formulated, exhibits the largest electroactive surface area, consequently offering a greater number of reactive sites.

The heterogeneous electron transfer rate constant (*k*_0_), which serves as a measure of the efficiency of electron transfer at the electrode–electrolyte interface, was determined from the EIS data using Equation S3.^34^ The *k*_0_ values for the bare GCE and the modified electrodes are summarized in Table 1. Among them, the AgCuAl-LDH/GCE exhibited the highest *k*_0_ value, indicating markedly improved charge transfer kinetics and superior electrochemical performance in the redox process. In addition, the standard exchange current density (j_0_), representing the intrinsic rate of the redox reaction under standard conditions, was calculated using Equation S4.^35^ As shown in Table 1, the j_0_ values demonstrate a pronounced enhancement in the electrocatalytic activity of the modified electrodes compared to the bare GCE. Given its outstanding performance, the AgCuAl-LDH/GCE was chosen for subsequent in-depth electrochemical investigation.

### 3.3 Parameters Optimization for the Modified Electrode

The optimization process began with the selection of an appropriate buffer solution. As shown in Figure S3A, various buffers, including acetate buffer, potassium chloride, phosphate-buffered saline, B-R buffer, hydrochloric acid, and sodium hydroxide, were evaluated through the application of DPV with 0.1 mM AFA. Among the tested conditions, the B–R buffer at pH 1.0 exhibited the most optimal response, yielding superior peak current and well-defined peak shape. Consequently, this buffer was chosen for all subsequent experimental analyses.

Afterward, the effect of different concentrations of the AgCuAl-LDH composite was evaluated over a range of 0.1–2.0 M (Figure S3B). The peak current reached its maximum at a concentration of 1.0 M. Beyond this point, increasing the composite concentration did not lead to any notable changes. Therefore, 1.0 M was selected as the optimal concentration for AFA determination.

As indicated in Figure S3C, different quantities of AgCuAl-LDH from 3.0 to 9.0 μL were applied onto the GCE surface. The most effective outcome was attained with 4.0 μL, where it reached its maximum response. Hence, a composite volume of 4.0 μL was chosen as the optimal amount for the experiment.

#### 3.3.1 Optimization of pH

The DPV analysis of AFA employing AgCuAl-LDH/GCE was explored across a pH range (1.0–6.0) in B-R buffer solution (Figure 8). Findings revealed that stable responses were exclusively achieved when measurements were conducted under acidic conditions. Notably, the most pronounced and well-defined peak was noted at pH 1.0. It was also remarked that at pH > 6.0, the anodic peak almost disappeared, which indicated the inert nature of AFA in a basic medium. Consequently, pH 1.0 was selected as the optimal pH value for AFA determination on the surface of AgCuAl-LDH/GCE.

**Figure 8:**
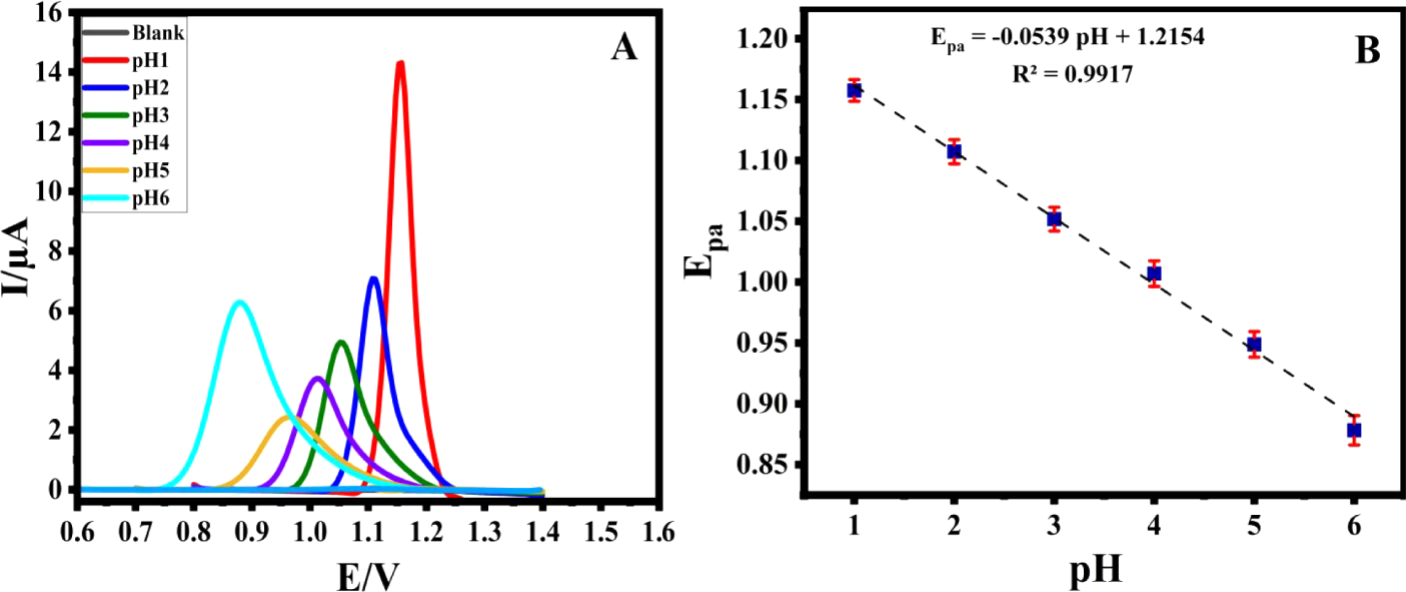
DPV recorded from the GCE modified with AgCuAl-LDH in B-R (pH 1.0) with 0.1 mM AFA at various pH (A) and the Ep versus pH value (B).

On the other hand, as the pH augmented, the peak potential of AFA shifted toward a more negative potential, and the relationship between peak potential and pH displayed a slope of 53.9 mV pH^–1^ (Figure 8). By applying this slope in the Nest Equation (eq S2),^36^ the ratio of protons and electrons involved in the oxidation process of AFA is calculated to be 0.914, which is approximately 1. Based on this finding, we suggest that the electrode process entails an equal participation of protons and electrons.^37^

### 3.4 Scan Rate Analysis

To study the oxidation kinetics of AFA on the AgCuAl-LDH/GCE, we conducted CV experiments in B-R buffer (pH 1.0) using scan rates (10.0–250.0 mV/s). When scan rates augment, the anodic peak currents (I_pa_) of AFA increase and exhibit a shift toward more positive potentials, as illustrated in Figure 9A.

**Figure 9:**
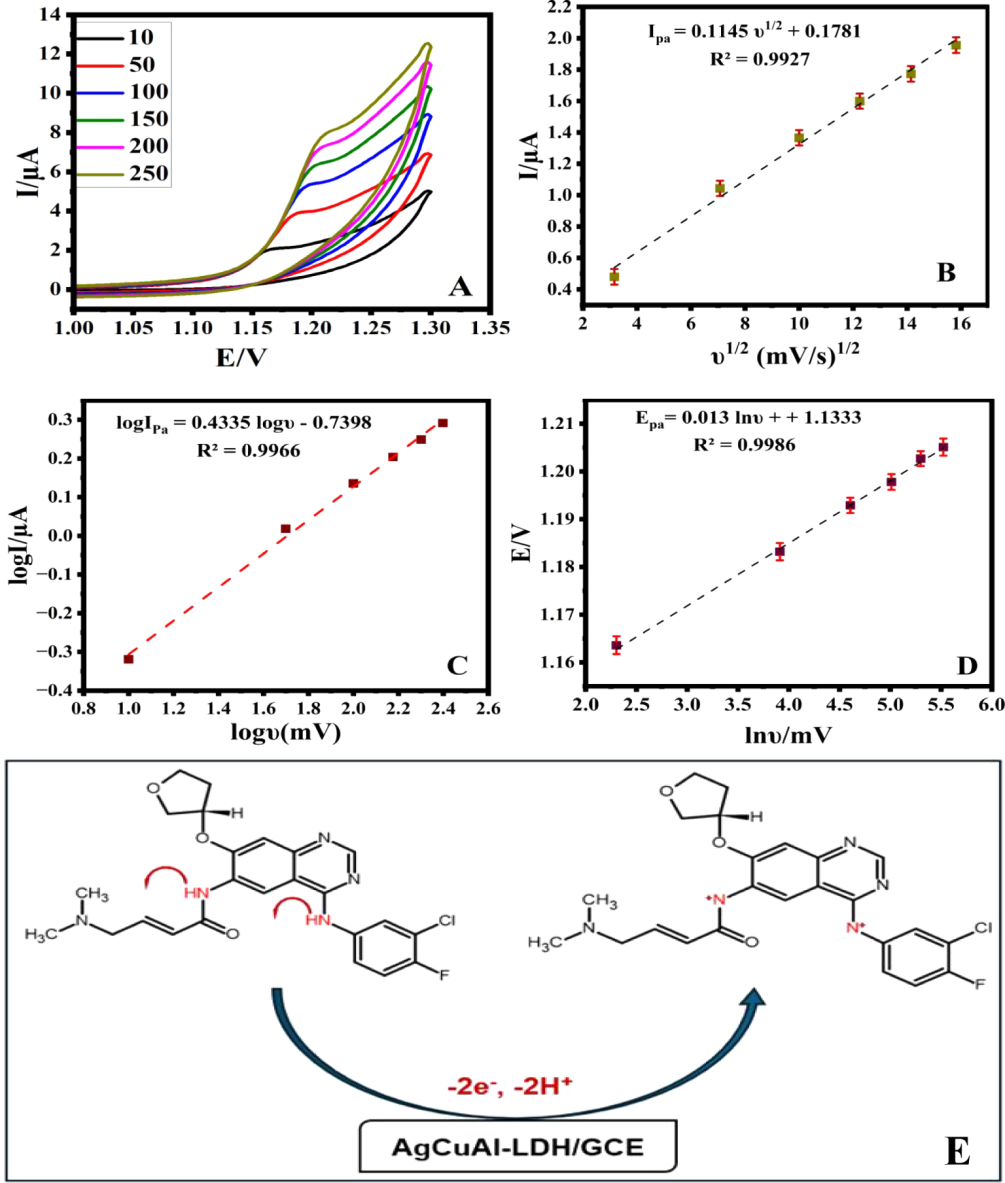
CV of 0.1 mM AFA in B-R (pH 1.0) recorded at various scan rates (A), the correlation between I_pa_ and υ^1/2^ (B), plots of log I_pa_ vs log υ (C), and E_pa_ vs ln υ (D) on the AgCuAl-LDH/GCE surface and possible oxidation reaction of AFA on AgCuAl-LDH/GCE (E).

Plotting I_pa_ against the square root of the scan rate (ν^1/2^) resulted in a linear relationship, as depicted in Figure 9B. This observation suggests that the charge transfer process is under diffusion control.^38^ Additionally, a linear correlation was observed between the logarithm of the anodic peak current (log I_pa_) and the logarithm of the scan rate (log v), yielding a slope of 0.433 (Figure 9C). This slope closely approximates the anticipated theoretical value of 0.5 for a diffusion-controlled process.^39^

A linear correlation between the anodic E_pa_ and the natural logarithm of the scan rate (ln ν) is illustrated by the equations depicted in Figure 9D. The calculation of the number of electrons transferred during the oxidation process of AFA was conducted employing the Laviron model, represented by the equation E_p_ = E° + [(2.303RT)/(1 – α)­nF] ln ν. After calculating the electron transfer coefficient (α = 0.53) by applying the slope of the Tafel curve (Figure S4) in the Tafel equation (2.3RT/n­(1 – α), the number of electrons participating in the AFA oxidation mechanism is equal to 2.0.

The findings of the pH study suggest that the electrochemical oxidation of AFA at the AgCuAl-LDH electrode proceeds through the transfer of 2 electrons and 2 protons. The potential reaction mechanism is illustrated in Figure 9E, as proposed in the previously mentioned study.^40^ This depiction highlights the interactions and processes that facilitate the oxidation of AFA, leveraging the unique properties of the AgCuAl-LDH composite on GCE.

### 3.5 Calibration

Figure 10A displays the peak currents obtained from the curves of DPV conducted on AgCuAl-LDH/GCE at various concentrations of AFA. The peak currents exhibit a linear increase corresponding to the rise in AFA concentration. A high correlation was noted between the peak current of AFA oxidation and its concentration across the range of 0.02 to 13.1 μM, yielding a high linear correlation coefficient of R^2^ = 0.9932 (Figure 10B). Based on the calibration curve, the lower detection limit (LOD) was determined to be 2.99 nM (3s/m), while the LOQ (10s/m) was found to be 9.96 nM (Table 2). “s” and “m” represent the standard deviation of peak current and slope of the calibration curve, respectively. Furthermore, the sensitivity of AgCuAl-LDH/GCE was calculated to be 1.65 μA·μM^–1^. cm^–2^ applying the equation below:

**Figure 10:**
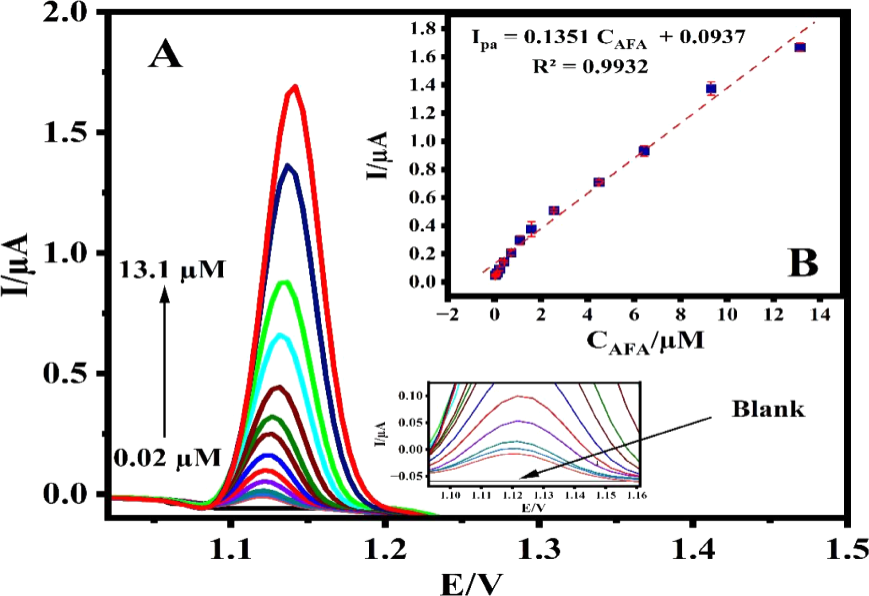
DPV of AFA concentrations ranging from 0.02 to 13.1 μM in B-R buffer (pH 1.0) on the surface of AgCuAl-LDH/GCE (A) and plot of the relationship between I_pa_ and the concentration of AFA (B).

**Table 2:** Analysis of Performance Metrics for the Proposed Method^a^

**Parameters**	**Value**
Intercept	0.09371 ± 0.01707
Slope	0.13509 ± 0.00338
n^a^	5
R^2^	0.9932
Linear range (μM)	0.02–13.1
LOD (nM)	2.99
LOQ (nM)	9.96
Sensitivity (μA·μM^–1^·cm^–2^)	1.65

a n: number of measurements

$Sensitivity=\frac{m}{a}$ where *m* is a slope of calibration curve (μA·μM^–1^) and *a* (cm^2^) is the surface area of AgCuAl-LDH/GCE.

To evaluate the analytical advantages of the proposed electrochemical method, its performance parameters were compared with those reported for established techniques such as RP-HPLC, UV–vis spectroscopy LC-MS/MS. The comparison, summarized in Table 3, considers key analytical figures of merit, including detection limit, linear dynamic range, analysis time, instrumentation cost, and operational complexity. While LC-MS/MS techniques typically achieve excellent sensitivity and selectivity, they require sophisticated instrumentation, prolonged sample preparation, and higher operational costs. In contrast, the AgCuAl-LDH/GCE-based electrochemical sensor demonstrates competitive sensitivity, a wide linear range, and rapid analysis capability, all within a simple and cost-effective platform. This highlights its suitability for routine analysis and on-site applications where rapid, low-cost detection is desirable.

**Table 3:** Comparison of the Proposed AgCuAl-LDH/GCE Electrochemical Sensor with Reported Methods for AFA Determination

**Methods**	**Real sample**	**Linear range**	**LOD**	**LOQ/LLOQ**	**ref**
LC-MS/MS	Human plasma	0.5–500 ng mL^–1^	0.42 ng	1.29 ng	^41^
LC-MS/MS	Human plasma	0.1–25.0 ng mL^–1^	-	0.10 ng·mL^–1^	^18^
RP-HPLC	Tablet	0.12–0.36 mg/mL	0.06 μg/mL	0.06 mg/mL	^42^
LC-MS/MS	Human serum	2–200 ng/mL	-	4.3 ng/mL	^43^
LC-MS/MS	Rat plasma	0.5–200 ng/mL	-	0.5 ng/mL	^44^
UV–vis spectroscopy	Bovine serum	0.3–10 μg mL^–1^	-	-	^45^
DPV	Tablet, Human urine and Human plasma	0.02–13.1 μM	2.99 nM	9.96 nM	Our work

### 3.6 Repeatability, Reproducibility and Stability

To assess the consistency of the GCE modified with AgCuAl-LDH, a sequence of 11 successive cycles was conducted, measuring the response for 10.0 μM of AFA (Figure S5A). The relative standard deviation (%RSD) was computed and calculated to be 2.96%, indicating remarkable repeatability of the AgCuAl-LDH/GCE.

Furthermore, to examine the reproducibility of the developed sensor, nine AgCuAl-LDH/GCE electrodes were assembled, and their respective current responses to a 10.0 μM AFA solution were assessed under identical conditions, utilizing repetitive DPV in B-R buffer (Figure S5B). The relative standard deviation (%RSD) across nine intraday experiments was calculated to be 2.19%, affirming the high reproducibility of the fabrication process.

Finally, as depicted in Figure S5C, the sensor’s stability was assessed by recording DPV curves over 2 two weeks. During the 2 weeks, the sensor was stored in a sealed container. DPV measurements were taken every 2 days using the AgCuAl-LDH modified GCE in a solution of 10 μM AFA within a B-R buffer at pH 1.0. The findings showed that the developed GCE retained 92.0% of its original current signal, reflecting the sensor’s excellent stability.

### 3.7 Selectivity

To investigate the selectivity of the AgCuAl-LDH/GCE electrochemical sensor, its response to 10.0 μM of AFA was examined in the presence of l-methionine, potassium chloride, sodium sulfate, l-arginine, sodium nitrate, dopamine, paracetamol, ascorbic acid, uric acid, d-glucose, urea, l-cysteine, and tyrosine. As demonstrated in Figure S6, even when the concentrations of interference products were increased to levels 100 times higher than that of AFA, the resulting signal variations were negligible, remaining below 5.0%. This suggests that these substances have negligible interference effects on the sensor’s response.

### 3.8 Real Samples

To evaluate its practical applicability, the AgCuAl-LDH-modified GCE was employed for the quantitative detection of AFA in various real-world samples, including commercial tablet formulations, human urine, and human plasma. For the biological samples, AFA was spiked into the matrices at known concentrations to assess the sensor’s recovery capability under realistic conditions. In the analysis of spiked urine samples, the method demonstrated excellent recovery rates, ranging from 95% to 98%, confirming its accuracy in complex biological environments. Similarly, when applied to tablet samples, the percentage recovery values were consistent and fell within a narrow range of 97% to 102%, reflecting the method’s robustness in pharmaceutical formulations. In the case of human plasma, which typically presents greater analytical challenges due to its complex composition, the recovery rates were slightly broader but remained acceptable, ranging from 96% to 112%. As detailed in Table 4, the %RSDs for these measurements were calculated to lie between 1.0% and 3.86%. These low %RSD values underscore the method’s high precision and reproducibility across different sample types, thereby validating the reliability and consistency of the AgCuAl-LDH/GCE sensor in diverse analytical applications.

**Table 4:** Determination of AFA in Real Samples

**Sample**	**Added (μM)**	**Found (μM)**	**RSD (%)**(*n* = 3)	**Recovery (%)**
**Tablet**	2.0	1.94	1.33	97.0
	6.0	5.92	2.00	98.7
	10.0	10.26	1.84	102.6
**Human urine**	2.0	1.95	2.85	95.0
	6.0	5.85	2.93	97.5
	10.0	9.80	3.14	98.0
**Human Plasma**	2.0	1.92	3.41	96.0
	6.0	6.04	2.89	100.6
	10.0	10.2	3.86	102.0

## 4 Conclusion

In this research, we have successfully fabricated an AgCuAl-LDH sensor capable of sensitively detecting AFA in various samples. The sensor exhibited excellent electrochemical performance, characterized by high sensitivity, stability, and selectivity toward AFA. Through various analytical methods, such as XRD and SEM, we proved the effective synthesis and structural properties of the AgCuAl-LDH. EIS further demonstrated the enhanced conductivity and efficient charge transfer capabilities of the nanocomposite.

The sensor’s performance was thoroughly evaluated under different pH conditions, with optimal sensitivity achieved at a specific pH level (pH 1.0). The repeatability and interference studies underscored the robustness and specificity of the sensor, making it a reliable tool for detecting AFA even in the presence of common interferents.

Real sample analysis, including pharmaceutical tablets, urine and human plasma, validated the sensor’s practical applicability, showing accurate and consistent results. The ability to retain 92% of its initial performance after 10 days of storage highlights the sensor’s long-term stability.

In conclusion, the AgCuAl-LDH sensor offers a highly effective and reliable method for the detection of AFA, with potential applications in environmental monitoring and clinical diagnostics. This research lays the groundwork for the continued advancement of sophisticated electrochemical sensors, which hold significant potential for use across diverse pharmaceutical and environmental fields.

## Supplementary Material



## Data Availability

Data will be made available on request.

## Notes

Additional experimental details, characterization data, and supplementary figures related to the optimization and validation studies (PDF)
