# Supplementary material for: A Silver–Copper–Aluminum Layered Double Hydroxide Sensor for Sensitive Determination of Anticancer Agent Afatinib in Bulk and Biological Fluids
Source: ACS Omega. 2025 Sep 30;10(40):47386–96. doi: 10.1021/acsomega.5c06746 (PMC12529382; doi:10.1021/acsomega.5c06746)
Supplement: Supplementary file 1 [file ao5c06746_si_001.pdf]

Supplementary Information

Silver-Copper-Aluminum Layer Double Hydroxide Sensor for Sensitive Determination of Anticancer Agent Afatinib in Bulk and Biological Fluids

Edoh Nicodème GABIAM[a], Nevin ERK[a], Mehmet Soner BAY[a], Asena Ayşe GENC[a], Hassan Elzain Hassan AHMED[b], Mustafa SOYLAK[b,c,d]

[a] Ankara University, Faculty of Pharmacy, Department of Analytical Chemistry, Ankara, Turkey; erk@pharmacy.ankara.edu.tr ; edohnicodemeg@gmail.com ; soner.bay@titck.gov.tr ; asenaaysegenc@gmail.com

[b] Erciyes University, Faculty of Sciences, Department of Chemistry, Kayseri, Turkey; wadelzain4891@gmail.com; msoylak@gmail.com

[c] Erciyes University, Technology Research & Application Center (TAUM), Kayseri, Turkey; msoylak@gmail.com

[d] Turkish Academy of Sciences (TUBA), Ankara, Turkey; msoylak@gmail.com

Chemicals and reagents

In this study, all chemicals were of analytical grade and employed without undergoing any additional purification. Aluminum nitrate nonahydrate (Al (NO3) 3 9H2O), Potassium hexacyanoferrate (III) (K3Fe(CN)6, 99.5 %), potassium chloride (KCl), hydrochloric acid (HCl) , Acetonitrile (CH3CN), sodium hydroxide (NaOH), sodium acetate, ascorbic acid, uric acid (99.0 %), acetic acid, sodium phosphate, Sodium sulfate (Na2SO4) were supplied by Merck Company (Darmstadt, Germany).

All experiments were conducted in a Britton-Robinson (B-R.) buffer, which consists of equimolar amounts of boric acid, phosphoric acid, and acetic acid. Additionally, 0.1 M sodium hydroxide was added to adjust the pH values. A 1:1 acetonitrile-water mixture was used to prepare the stock solution of Alectinib. Distilled water was used throughout.

Apparatus

XRD analysis of the prepared materials was carried outusing Rigaku Rint 2000 X-ray Diffractometer between 2 and 80 /min with a scan rate of 2/min.

The specimens were investigated by JEOL JSM 6510LV scanning electron microscope (SEM) at 15 kV. The morphology at nanoscale was further observed by JEOL JEM 2100 High Resolution Transmission Electron Microscope (LaB6 filament) operated at 200 kV and equipped with an Oxford Instruments X-Max 80T Energy Dispersive Spectrometer (EDS) system. Carbon support film coated copper TEM grids (Electron Microscopy Sciences, CF200-Cu, 200 mesh) were used. Images were taken by Gatan Model 833 Orius SC200D CCD Camera. HRTEM images were taken by by Gatan Model 794 Slow Scan CCD Camera. Gatan Microscopy Suite (GMS) 2 software was used. For diffraction pattern indexing CrystBox software was used [M. Klinger. CrysTBox - Crystallographic Toolbox. Institute of Physics of the Czech Academy of Sciences, Prague, 2015. ISBN 978-80-905962-3-8. URL http://www.fzu.cz/~klinger/ crystbox.pdf]. The elemental composition and phase structure were analyzed by X-ray photoelectron spectroscopy (XPS) were recorded using a Specs-Flex XPS instrument in the range of 200-4000 eV.

Voltametric experiments were conducted using an AUTO LAB system equipped with a PGSTAT204 electrochemical workstation (Metrohm Inc., Switzerland). Throughout the

experiments, a standard three-electrode system was employed in a one-compartment of 10 mL electrochemical cell. AgCuAl-LDH/GCE, a platinum rod, and Ag/AgCl were used as the working, counter, and reference electrodes, respectively. The pH values of the prepared Britton-Robinson (B-R) buffer solutions were determined using a HANNA pH meter (edge® Multiparameter pH Meter - HI2020).

The Randles-Sevcik equation:

$$I = (2.69 \times 10^5)\, n^{\frac{3}{2}}\, A\, D^{\frac{1}{2}}\, v^{\frac{1}{2}}\, C_0 \quad (\mathbf{S1})$$

$$E_{eq} = E^{\circ} + \frac{0.0592}{z} \lg a_{H^+}^{m} = E^{\circ} - 0.0592\frac{m}{z}\, pH \quad (\mathbf{S2})$$

(3) where E° (V) is the standard potential, a (mol·L−1) is the activity, m and z are the numbers of protons and electrons taking part in the reaction, respectively.

$$R_{ct} = \frac{RT}{F^2\, C\, A\, k^0} \quad \textbf{(S3)}$$

$$R_{ct} = \frac{RT}{n\, F\, A\, j_0} \quad \textbf{(S4)}$$

In this context, $\mathbf{k_0}$ denotes the standard heterogeneous electron transfer rate constant ($cm \cdot s^{-1}$), while $\mathbf{j_0}$ refers to the exchange current density ($A \cdot cm^{-2}$). **R** represents the universal gas constant (8.314 $J \cdot K^{-1} \cdot mol^{-1}$), **T** is the absolute temperature (298.15 K), and **F** is the Faraday constant (96,485 $C \cdot mol^{-1}$). **Rct** corresponds to the electron transfer resistance (Ω), **A** is the geometric surface area of the electrode (cm^2), **n** is the number of electrons involved in the redox process, and **C** is the concentration of the $[Fe(CN)_6]^{3-/4-}$ solution (5×10^{-6} $mol \cdot cm^{-3}$).

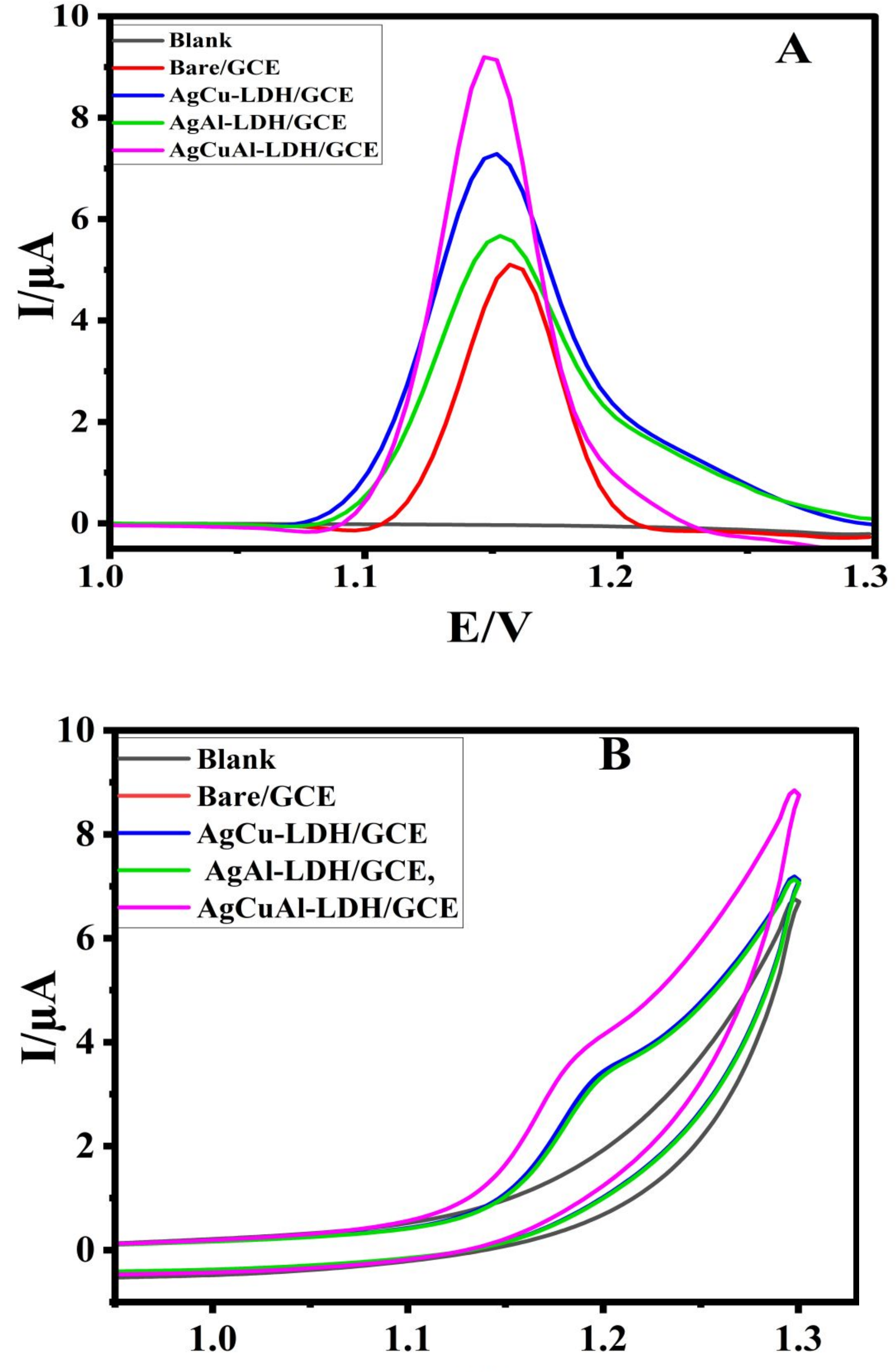

Figure S1. CV at 50.0 mVs^{-1} (A) and DPV (B) of AFA (0.1mM) in 0.1M of B-R at Bare/GCE, AgCu-LDH/GCE, AgAl-LDH/GCE, and AgCuAl-LDH/GCE.

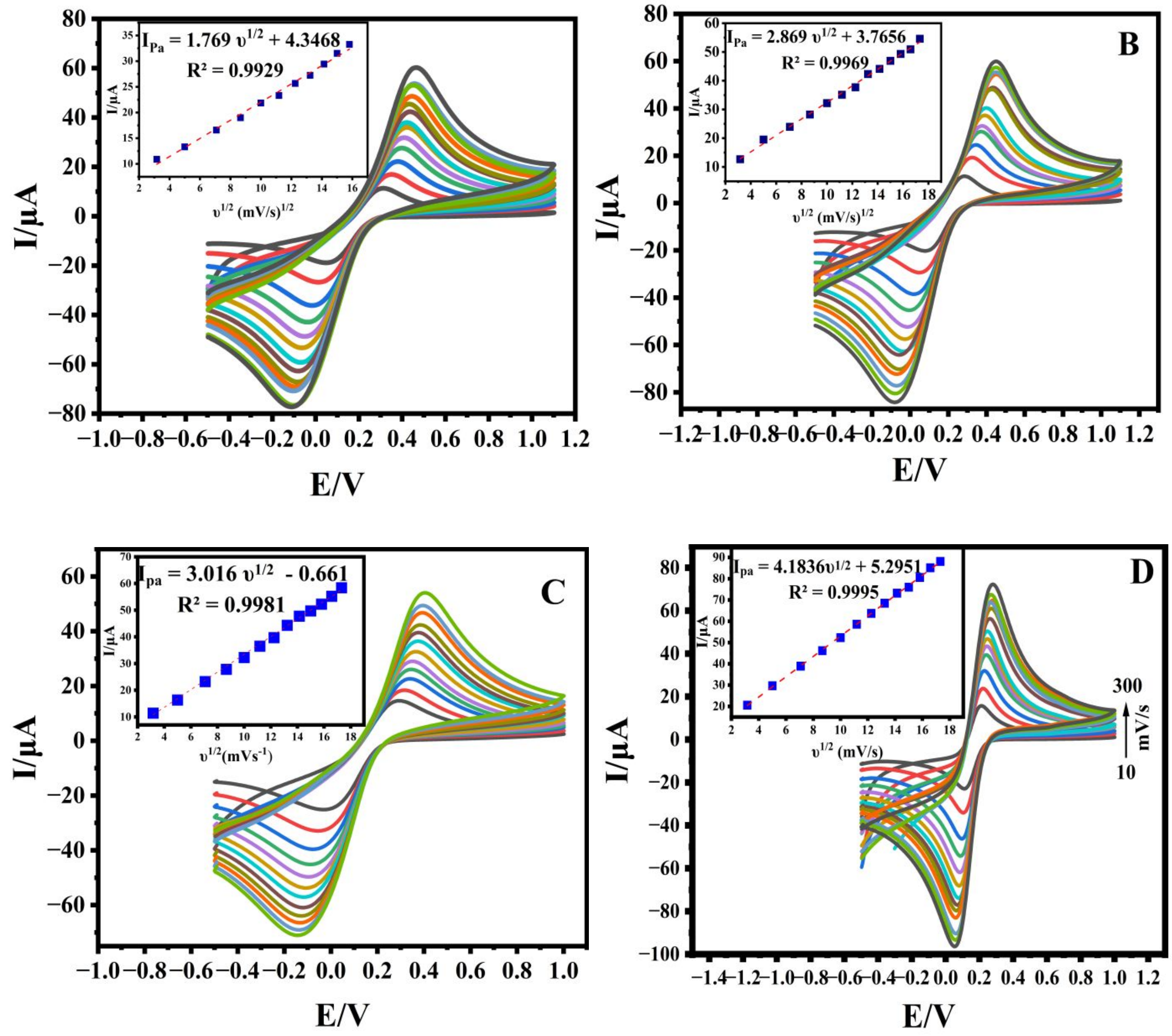

Figure S2. The recorded CV curves at various scan rates in the presence of 5.0 mM [Fe (CN) 6]3−/4− containing 0.1 M KCl on bare GCE (A), AgCu-LDH/GCE (B), AgAl-LDH/GCE (C) and AgCuAl-LDH/GCE (D)

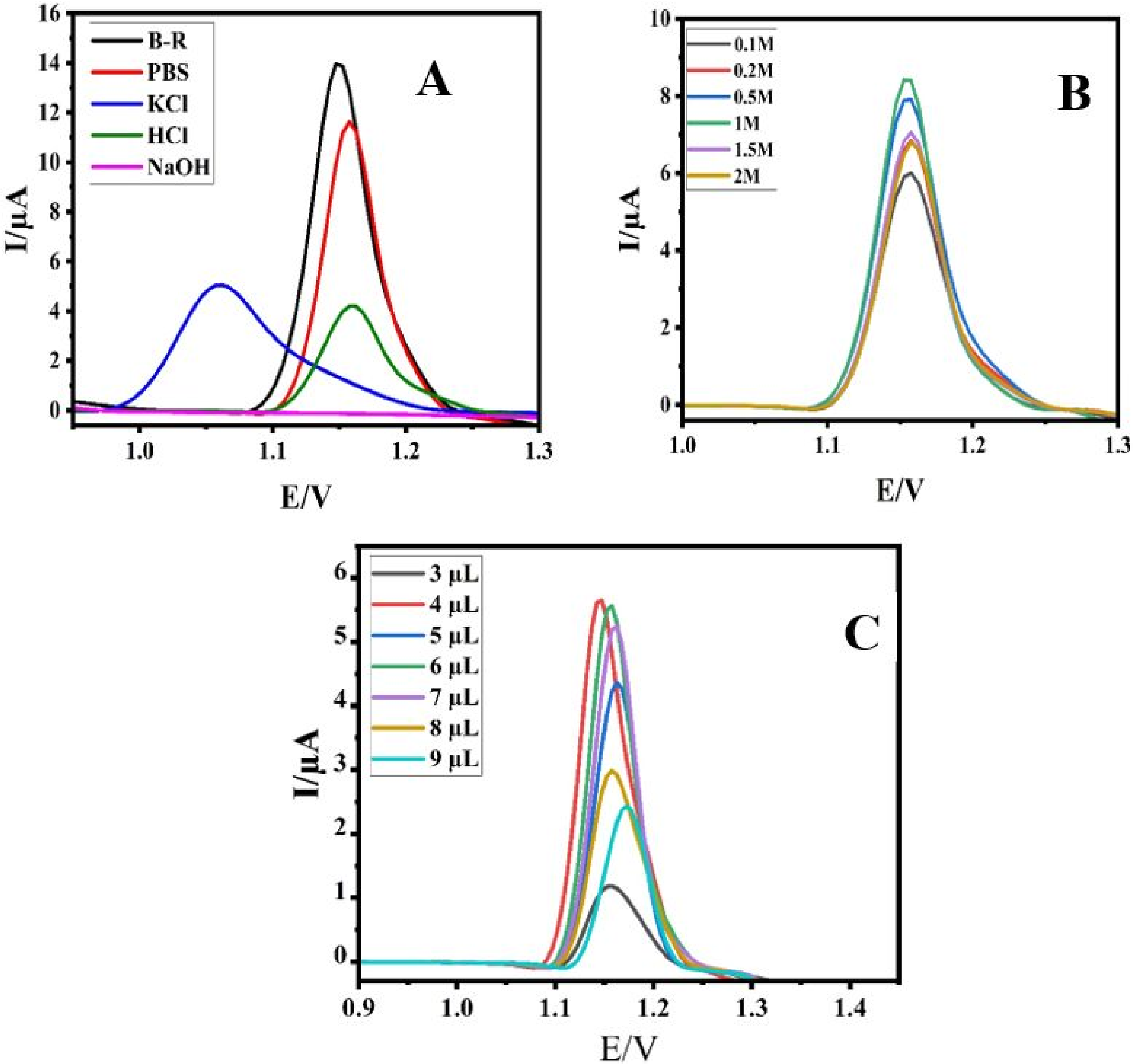

Figure S3. Influence of supporting electrolyte (A), the concentration (B), and the amount (C) of AgCuAl-LDH composite on the oxidation peak currents of 0.1 mM AFA.

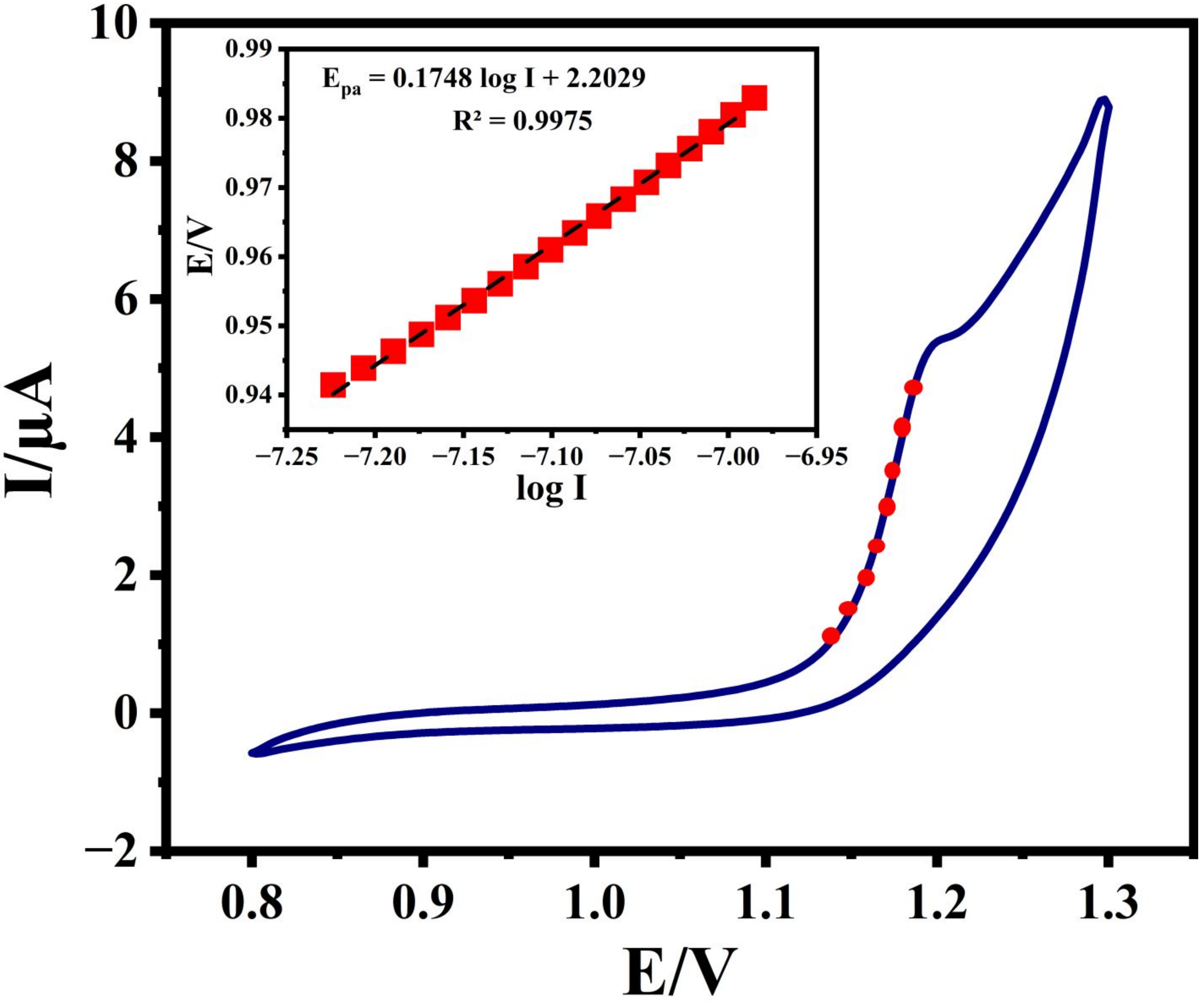

Figure S4. Tafel plot for 0.1 mM AFA with scan rates of 100 mVs^{-1} on the surface of AgCuAl-LDH/GCE.

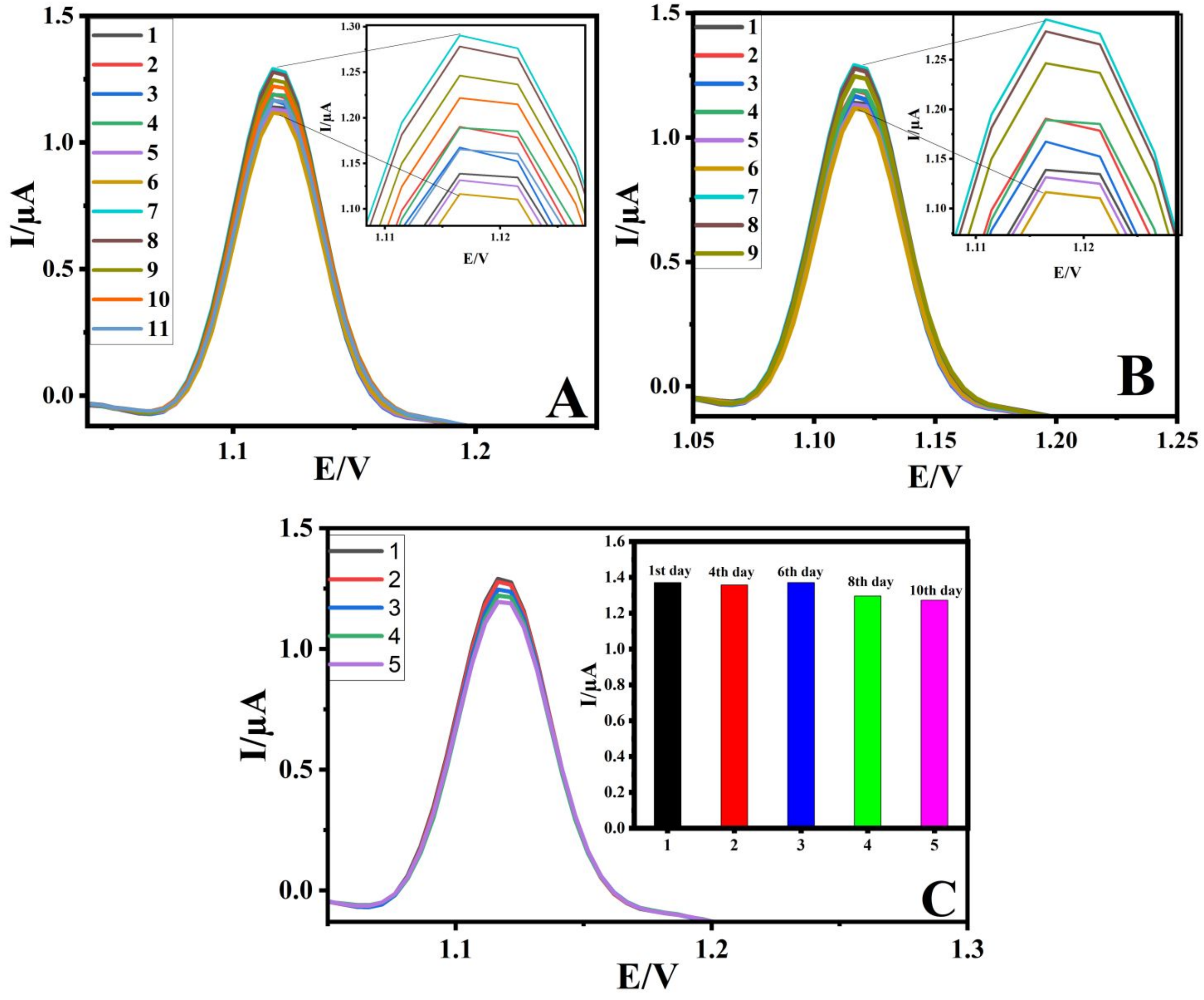

Figure S5. Repeatability (A), reproducibility (B), and stability (C) of 10.0 μM AFA at AgCuAl-LDH/GCE in B-R buffer (pH 1.0).

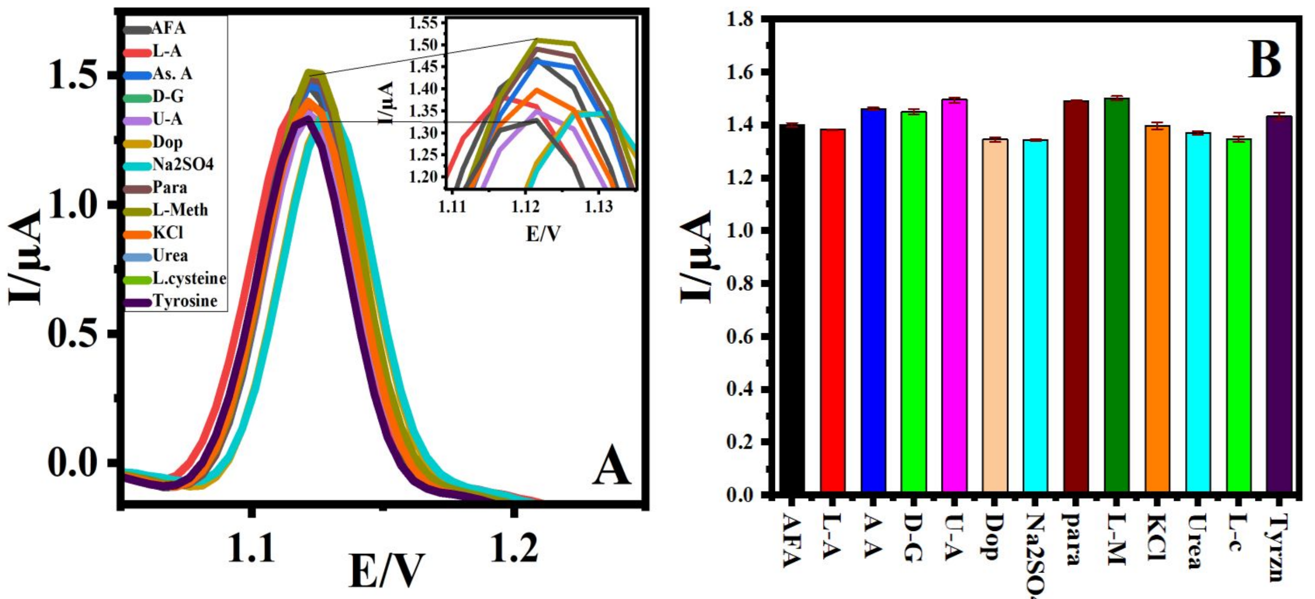

Figure S6. Interference study of 10.0 μM AFA at AgCuAl-LDH/GCE in B-R buffer (pH 1.0)
